# MR imaging diagnosis of small-cell carcinoma of the ovary, hypercalcemic type: A case report and literature review

**DOI:** 10.1097/MD.0000000000039226

**Published:** 2024-08-09

**Authors:** Xinyu Li, Zhuolin Liu, Jiake Chen, Huifen Hao, Dongmei Fan, Wenbin Huang

**Affiliations:** a Imaging Center, The First Affiliated Hospital, College of Clinical Medicine of Henan University of Science and Technology, Luoyang, China; b Gynecology Ward III, the First Affiliated Hospital, College of Clinical Medicine of Henan University of Science and Technology, Luoyang, China; c Pathology Department, the First Affiliated Hospital, College of Clinical Medicine of Henan University of Science and Technology, Luoyang, China.

**Keywords:** MRI, ovarian tumor, SCCOHT

## Abstract

**Rationale:**

Small-cell carcinoma of the ovary, hypercalcemic type (SCCOHT), is a rare and aggressive gynecological tumor. We retrospectively analyzed the clinical manifestations and imaging findings of this patient and analyzed the relevant literature, with the aim of improving the ability of radiologists to differentiate SCCOHT from other ovarian tumors.

**Patient concerns:**

We report a case of 36-year-old woman who was diagnosed with SCCOHT. MRI suggested a malignant tumor of the left ovary. The immunohistochemical markers shows SMARCA4 negativity. Notably, hypercalcemia was not detected. Microscopically, it was consistent with the large-cell variants.

**Lessions:**

Despite its rarity, SCCOHT should still be considered in the differential diagnosis of ovarian malignancies. When a young female patient presents with a large unilateral tumor on MRI with a predominant solid component and significant enhancement on the contrast enhanced scans, along with hypercalcemia, SCCOHT should be considered.

## 1. Introduction

Small-cell carcinoma of the ovary, hypercalcemic type (SCCOHT) is an undifferentiated tumor composed of small cells.^[1]^ To date, fewer than 500 cases have been reported in the literature.^[2]^ SCCOHT accounts for less than 0.01% of gynecologic malignancies, with peak incidence in young adulthood (range 0–55 years; median age: 24 years).^[2]^ Patients with SCCOHT often exhibit elevated level of human epididymis protein (HE4) and CA-125, and approximately two-thirds of patients present with hypercalcemia. SMARCA4(BRG1)-mutant is considered a pathogenic mutation leading to the pathogenesis of SCCOHT.

Here we present the case of a 36-year-old female patient diagnosed with SCCOHT, who did not exhibit hypercalcemia and microscopic examination revealed the presence of the large-cell variants.

## 2. Case presentation

A 36-year-old woman presented with a chief complaint of an irregular menstrual cycle with reduced menstrual flow for over 6 months. Additionally, she reported the presence of a pelvic mass for 7 days, without any symptoms of abdominal distension or pain. Seeking treatment, she visited our hospital, where an ultrasound examination identified a cystic-solid mass in the upper left quadrant of the uterus, predominantly solid in nature. Physical examination revealed a soft, approximately 6 cm mass in the left adnexal area, demonstrating good mobility and no tenderness. No palpable anomalies were noted in the right adnexal area. Tumor marker analysis revealed a significantly elevated CA-125 level at 190.81 U/ml, and an elevated AFP level at 14.17 ng/ml. The assay for vascular endothelial growth factor yielded a value of 231.52 pg/ml, and her serum calcium level was measured at 2.24 mmol/l (normal < 2.75 mmol/l).

**Figure 1.**
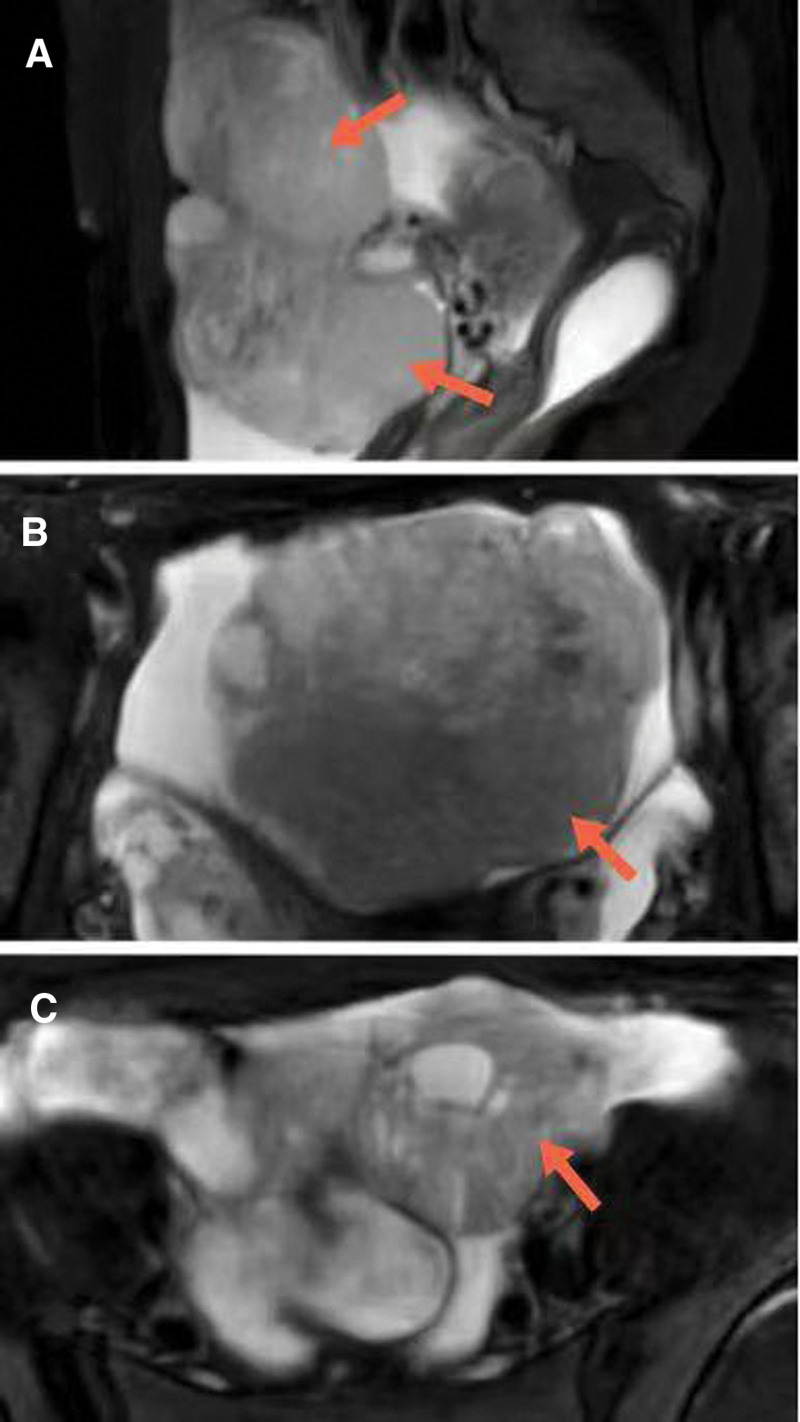
(A–C) A 36-year-old female patient with small cell carcinoma of the ovary, hypercalcemic type. Axial and sagittal T2-weighted MR images demonstrate a heterogeneous mass.

**Figure 2.**
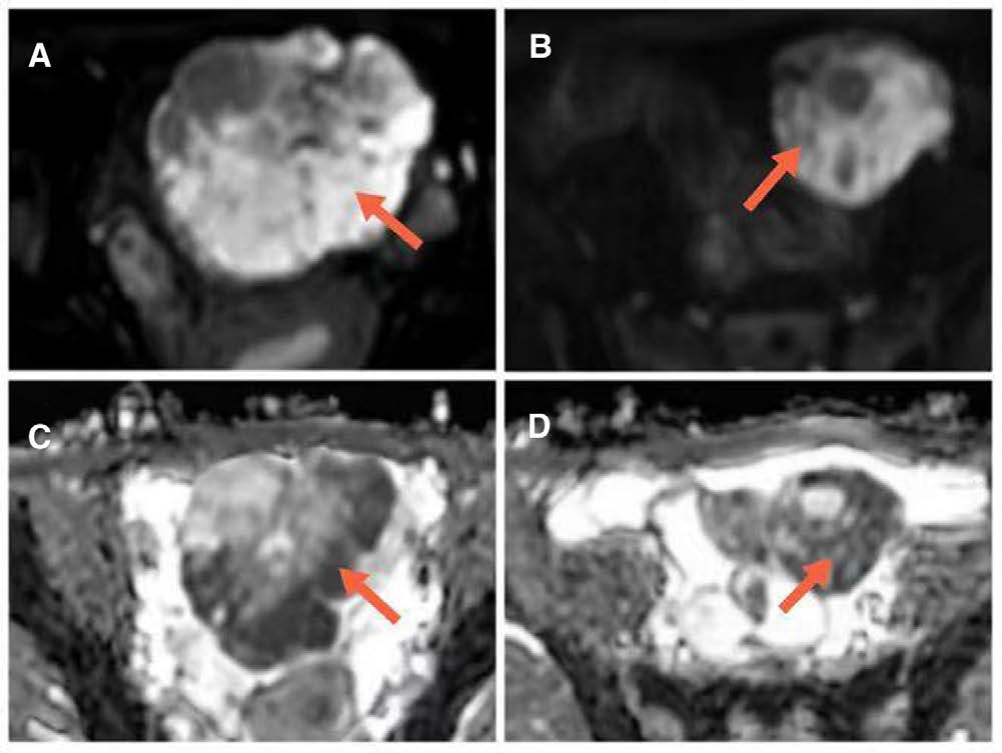
(A and B) When *b* = 800s/mm^2^, the solid component showed a slightly hyperintense signal on DWI images. (C and D) ADC images showed a hypointense signal.

**Figure 3.**
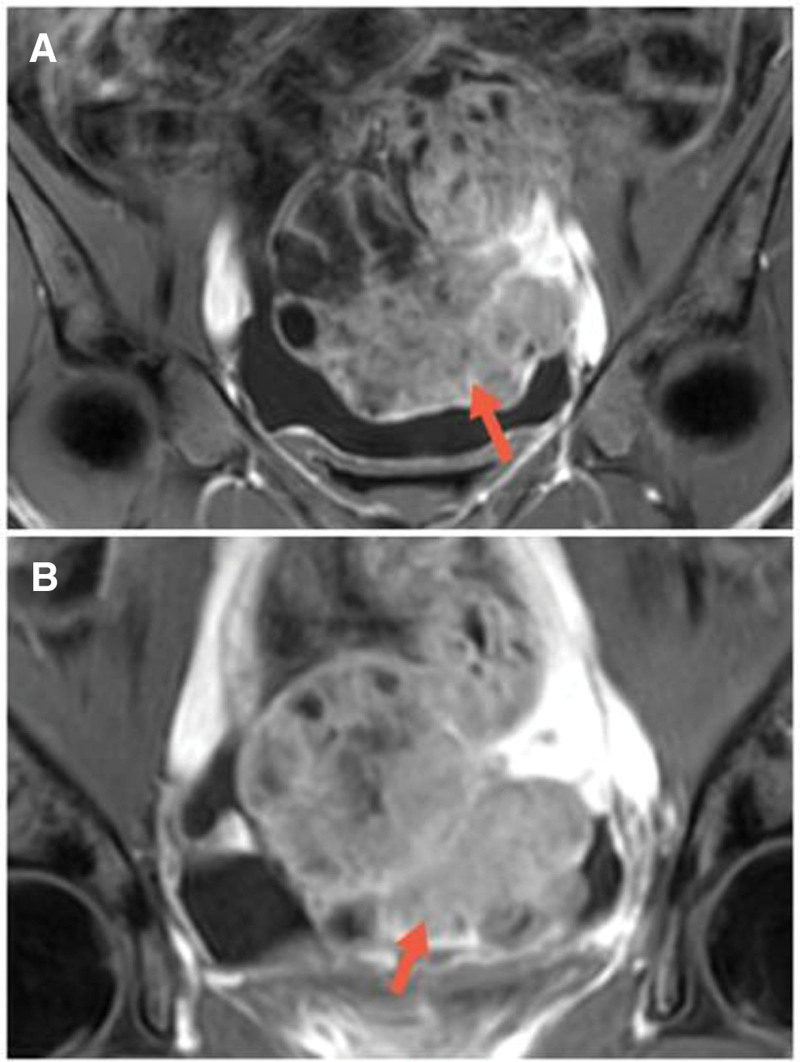
(A and B) The septa and edges of the lesions were enhanced and the solid part was significantly enhanced.

Magnetic Resonance Imaging (MRI) images demonstrate a large heterogeneous mass (105 × 68 × 88 mm) with a large amount of solid component and cystic changes in a focal area. The tumor showed heterogeneous hypointensity on T1-weighted imaging. On the T2WI, the tumor displays heterogeneous hypointensity mixed a small amount of high T2 signal (Fig. 1). On the axial Diffusion Weighted Imaging (DWI) and correlative Apparent Diffusion Coefficient (ADC) map, the tumor displays hyperintensity and hypointensity, respectively (Fig. 2). The contrast-enhanced MRI revealed significant enhancement of the solid part (Fig. 3). Free pelvic fluid was also observed.

**Figure 4.**
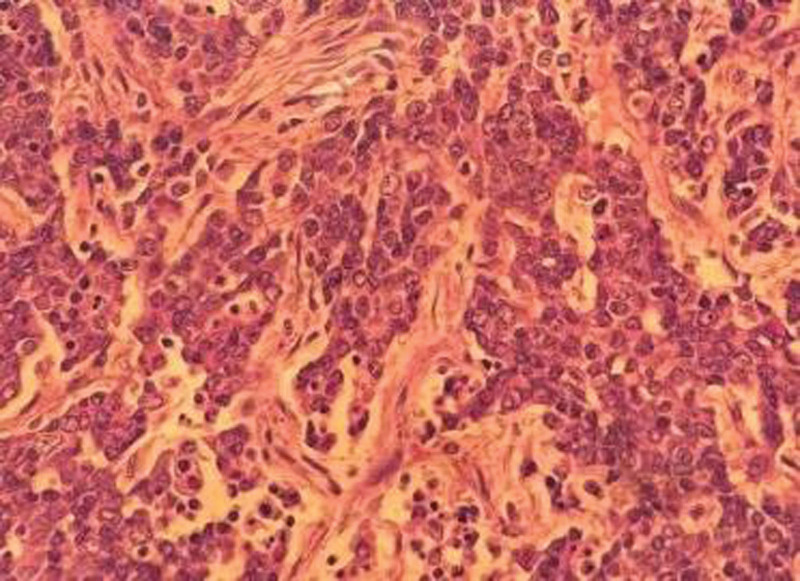
The tumor cells were infiltrative and varied in size, a glassy eosinophilic cytoplasm and eccentric large pale nuclei are to be seen, indicating the presence of large-cell variants.

Since the tumor was initially diagnosed as a dysgerminoma, the patient underwent a left adnexectomy for the first time, after the pathological diagnosis of SCCOHT. Given that the patient has no further reproductive needs, the patient then received right adnexectomy, total hysterectomy and lymph node dissection. During the operation, a bunch of gray-red and gray-white broken tissue was observed, with gray-yellow cut surface and soft texture. The excised tissue was submitted for pathological examination, diffuse small and big, round cells with scant cytoplasms, a glassy eosinophilic cytoplasm and eccentric large pale nuclei, hyperchromatic nuclei, and active nuclear divisions were detected in the microscopy (Fig. 4). The immunohistochemical staining results were as follows: CK(AE1/AE3)-focal positive, C30-negative, WT-1-positive, SALL-4-positive, Ki-67: about 40% (+), ER-negative, SMARCA4-negative, INI1-positive, S-100-negative, Syn-weak positive, Desmin-negative, C45(LCA)-negative, C43-negative, 0K8/18-foval positive, CK7-negative, inhib in-a-negtive. Notably, the immunohistochemical markers showed slight positivity, but it is important to highlight the SMARCA4 negativity.

## 3. Discussion

SCCOHT is an extremely rare ovarian malignancy with a poor prognosis. It primarily affects young women with a mean age of 22 years and usually involves unilateral ovarian involvement. The disease typically occurs unilaterally, with a higher incidence on the right side.^[2]^ In the 2014 World Health Organization Classification of Tumours of Female Reproductive Organs, SCCOHT was classified as a miscellaneous neoplasm.^[3]^ Approximately 36.4% to 60% of the patients have hypercalcemia at the time of diagnosis,^[1]^ which is believed to be due to the secretion of parathyroid hormone-related peptide by the tumor, and some patients may even have hypercalcemic-related symptoms such as nausea, vomiting, polydipsia, and polyuria. However, hypercalcemia was not observed in this case.

Histologically, the gross specimen of SCCOHT in this case displayed a gray-red and gray-white cut surface with a soft texture. Microscopically, it was mainly characterized by monomorphic small cells and follicular structures, however, in this case, glassy eosinophilic cytoplasm and eccentric large pale nuclei were detected in the microscopy. Some studies have found that all SMARCA4-negative SCCOHTs also lack SMARCA2 protein by IHC. SMARCA4 loss, either alone or in combination with SMARCA2, has high sensitivity and specificity in the diagnosis of SCCOHT.^[4]^ In this case, loss of SMARCA4 (BRG1) expression was confirmed by immunohistochemistry, leading to the diagnosis of SCCOHT.

On imaging, SCCOHT typically presents as a large tumor that is usually unilateral, with a predominant solid component and significant enhancement on enhanced scans. Hemorrhage, necrosis, and cystic degeneration may occur in the center of the lesion. However, it is difficult to directly diagnose SCCOHT based on its imaging findings alone. For the diagnosis of this disease, clinical information such as the age of onset and relevant laboratory tests should be combined to help us in the differential diagnosis. The following cystic-solid masses with predominantly solid components should be distinguished.

Dysgerminoma of the ovary: It is the most common ovarian malignant germ cell tumor, accounting for 32.8% to 37.5% of all ovarian malignant germ cell tumors.^[5]^ It is more common in young women and usually occurs in the right ovary. The classic appearance of dysgerminoma is that of a large, well-defined solid adnexal mass composed of multiple lobules with intervening fibrovascular septations, and a prominent vascular pedicle with tortuous vessels.^[6]^ Enhancement was heterogeneous, and the degree of enhancement was usually less than myometrium. Because of the fibrous content, the septa appear as hypointensity on T2WI and may show significant enhancement on contrast-enhanced Magnetic Resonance (MR) images.^[7]^ It is noteworthy that dysgerminoma is usually accompanied by the elevation of serum lactate dehydrogenase, usually isoenzymes 1 and 2.^[8]^

Yolk sac tumors: Yolk sac tumors are most commonly encountered in women in the second and third decades of life and are rare in women over 40 years. They are characterized by rapid growth and poor prognosis.^[9]^ In laboratory examination, elevated AFP is of great diagnostic significance. In addition, studies have shown that ZBTB16 is a sensitive and specific marker for yolk sac tumors and is diagnostically superior to AFP.^[10]^ On MRI, yolk sac tumors usually appear as large cystic-solid masses with smooth margins, the bright dot sign is a common finding and is seen at contrast-enhanced MR imaging as enhancing foci in the wall or solid components.^[11]^ On T1WI, areas of hemorrhage exhibit high intensity, which is important to consider for yolk sac tumor in relatively young women. On T2WI, the tumor exhibits heterogeneous high intensity, reflecting edematous stroma, cysts, and hemorrhage.^[12]^

Sclerosing stromal tumor of the ovary: Sclerosing stromal tumor of the ovary is a rare benign sex cord-stromal tumor of the ovary, which occurs predominantly in the second and third decades of life.^[13]^ Relevant studies have shown that inhibin and calretinin are important immunohistochemical markers, which are helpful for the diagnosis of sclerosing stromal tumor of the ovary.^[14]^ Radiologically, T1WI displayed hypointensity, and T2WI showed mixed hyperintensity, the cystic area in the center of the lesion showed significant hyperintensity, that is, “lake-island” sign. The outer surface of the tumors had a complete capsule and smooth margin.^[15]^ The enhancement showed progressive, continuous, and centripetal enhancement, with enhanced comb wall nodules.

In terms of treatment and prognosis, due to the rarity of SCCOHT, there is currently no standard therapy for SCCOHT. The treatment options for SCCOHT are very diverse and may include surgery, chemotherapy, radiotherapy and autologous stem cell transplantation following high-dose chemotherapy.^[16]^ Surgery combined with adjuvant chemotherapy is the most widely used approach. The most common surgical methods in surgical treatment are unilateral salpingo-oophorectomy and total abdominal hysterectomy with bilateral salpingo-oophorectomy. SCCOHT primarily affects young females and there is currently no definitive conclusion regarding whether fertility-sparing surgical procedures should be adopted. Based on the available research data both domestically and internationally, fertility preservation surgeries do not appear to significantly impact survival benefits. Considering the high malignancy of the disease, radical surgery may potentially lower the risks of recurrence and metastasis.^[17]^ In addition to that, genetic counseling is recommended for women affected by SCCOHT. Annual control with ultrasonography and Ca/parathyroid hormone-related peptide levels is recommended.^[2]^ Prophylactic oophorectomy can be considered for elderly SMARCA4 mutation carriers.^[18]^

## 4. Conclusion

Despite its rarity, SCCOHT should still be considered in the differential diagnosis of ovarian malignancies. When a young female patient presents with a large unilateral tumor on MRI with a predominant solid component and significant enhancement on the contrast enhanced scans, along with hypercalcemia, SCCOHT should be considered. However, definitive diagnosis still requires pathological examination and immunohistochemistry testing. To date, surgery combined with adjuvant chemotherapy is the most widely used treatment approach.

In summary, the purpose of reporting this case is to familiarize radiologists with the clinical features and imaging manifestations of SCCOHT, by increasing recognition and enhancing preoperative differential diagnostic capabilities, we aim to improve patient care and outcomes.

## Author contributions

Conceptualization: Xinyu Li.

Data curation: Xinyu Li, Zhuolin Liu, Jiake Chen, Huifen Hao, Dongmei Fan, Wenbin Huang.

Formal analysis: Xinyu Li.

Funding acquisition: Xinyu Li.

Investigation: Xinyu Li, Jiake Chen, Huifen Hao, Dongmei Fan, Wenbin Huang.

Resources: Jiake Chen, Huifen Hao.

Visualization: Xinyu Li.

Writing-original draft: Zhuolin Liu.
